# Deep Learning in Multi-Class Lung Diseases’ Classification on Chest X-ray Images

**DOI:** 10.3390/diagnostics12040915

**Published:** 2022-04-06

**Authors:** Sungyeup Kim, Beanbonyka Rim, Seongjun Choi, Ahyoung Lee, Sedong Min, Min Hong

**Affiliations:** 1 Department of Software Convergence, Soonchunhyang University, Asan 31538, Korea; sungyeup.kim@gmail.com (S.K.); rim.beanbonyka@sch.ac.kr (B.R.); 2 Department of Otolaryngology-Head and Neck Surgery, Cheonan Hospital, Soonchunhyang University College of Medicine, Cheonan 31151, Korea; akas9238@hanmail.net; 3 Department of Computer Science, Kennesaw State University, Marietta, GA 30144, USA; alee146@kennesaw.edu; 4 Department of Medical IT Engineering, Soonchunhyang University, Asan 31538, Korea; sedongmin@sch.ac.kr; 5 Department of Computer Software Engineering, Soonchunhyang University, Asan 31538, Korea

**Keywords:** multi-class classification, deep learning, transfer learning, EfficientNet v2, chest X-ray image

## Abstract

Chest X-ray radiographic (CXR) imagery enables earlier and easier lung disease diagnosis. Therefore, in this paper, we propose a deep learning method using a transfer learning technique to classify lung diseases on CXR images to improve the efficiency and accuracy of computer-aided diagnostic systems’ (CADs’) diagnostic performance. Our proposed method is a one-step, end-to-end learning, which means that raw CXR images are directly inputted into a deep learning model (EfficientNet v2-M) to extract their meaningful features in identifying disease categories. We experimented using our proposed method on three classes of normal, pneumonia, and pneumothorax of the U.S. National Institutes of Health (NIH) data set, and achieved validation performances of loss = 0.6933, accuracy = 82.15%, sensitivity = 81.40%, and specificity = 91.65%. We also experimented on the Cheonan Soonchunhyang University Hospital (SCH) data set on four classes of normal, pneumonia, pneumothorax, and tuberculosis, and achieved validation performances of loss = 0.7658, accuracy = 82.20%, sensitivity = 81.40%, and specificity = 94.48%; testing accuracy of normal, pneumonia, pneumothorax, and tuberculosis classes was 63.60%, 82.30%, 82.80%, and 89.90%, respectively.

## 1. Introduction

Lung disease is one of the top causes of death worldwide and includes pneumonia, pneumothorax, and tuberculosis diseases [1]. For example, pneumonia is a disease that causes air sacs in the lungs [2]. Its symptoms include fever, cough, shortness of breath, loss of appetite, stabbing chest pain, low energy, vomiting, and confusion, especially in older people. Pneumothorax is a disease that can cause complete or partial lung collapse [3]. The lung collapse can potentially be caused by intense activities such as mountain climbing, flying, or scuba diving. Those intense activities may cause huge changes in air pressure. Symptoms of pneumothorax commonly include chest pain, shortness of breath, rapid breathing, a dry and hacking cough, bluish skin, and fatigue. Tuberculosis is a disease provoked by an airborne bacterial infection in the lung [4]. Symptoms of tuberculosis include a cough for three consecutive weeks, a loss of appetite that yields unintentional weight loss, chills, fever, and night sweats.

Therefore, early assessment and diagnosis can significantly reduce the life-threatening nature of lung diseases and improve the quality of life of suffering patients [5,6,7,8]. In modern medical image modalities, imaging tests are extremely powerful tools that can help doctors diagnose a range of conditions. The most commonly used image modalities are the chest X-ray radiographic image (CXR) and computed tomography (CT). They are diagnostic tools that allow doctors to see the internal structures of the body without cutting.

Lung disease detection is currently performed through an examination of CXR images by a professional radiologist, due to its convenient and non-invasive assessment for overall findings of the chest situation in brief. It is also suitable for follow-up examination since disease changes can be observed more easily and earlier. However, there is a common human error that may be caused by the misreading of a CXR image due to the complex anatomical structure of the chest. Therefore, computer-aided diagnostic systems (CADs) are used to help radiologists overcome clinical decisions with more precise diagnosis and to minimize misreading. To improve the efficiency and accuracy of the diagnostic performance of CADs, the CXR image has been widely exploited by various methods.

The deep learning (DL) method has outperformed traditional machine learning in medical image analysis on classification, segmentation, and detection tasks [5,8], which apply to various data modalities ranging from a 1D signal [9,10], 2D [11,12,13] to a 3D image [14]. Compared to the natural RGB image, the CXR image is a grayscale image with one-channel information. The information of the CXR image is scanned by an X-ray, also called a radiograph [15]. The X-ray sends radiation through the body. Areas with high levels of calcium (i.e., bones) block the radiation, causing them to appear white on the image. Soft tissues (i.e., lungs, heart, liver, muscle, and more) allow the radiation to pass through and appear in a range from gray to black on the image. The CXR image is commonly stored as a one-channel grayscale file in png [16] or tiff format [17], which causes a unique challenge for the DL method. Some research applied the DL model to the one-channel grayscale CXR image by learning from scratch (with random, initialized weights), which yielded limited results [18]. To overcome this limitation, an intuitive method is to collect more data. However, both the collection and labeling of medical data require experts and consume a lot of time and money. Some other research converted the one-channel grayscale image to a three-channel image by adding its information three times repeatedly [19,20,21]. Since the convolutional neural network (CNN) is the most significant DL model for 2D image classification, it can learn and distinguish on a three-channel image more precisely than on a one-channel image. However, the three-channel, converted grayscale image has no new information. Therefore, by training the DL model on it via learning from scratch still yielded limited results. The latest solution on a three-channel, converted grayscale image was to apply a transfer learning technique [11,22,23,24,25,26,27] by transferring the learned weights of a model trained on a three-channel natural RGB image and then fine-tuning the model on a three-channel, converted grayscale image. Although the CXR image is far different from the natural RGB image, the model trained on the RGB image may extract more general features, which can help to identify shapes, patterns, edges, and object composition. These similar features may also be effective for classifying lung diseases.

Therefore, in this paper, we propose the DL method using a transfer learning technique to classify lung diseases in CXR images to improve the efficiency and accuracy of a CADs’ diagnostic performance.

Our main contributions are as follows:

- We exploited the convolutional neural network (CNN) model in general (EfficientNet v2-M model in specific) to classify lung diseases using transfer learning techniques with empirical hyperparameters to achieve a significant performance;
- Our proposed method was a one-step, end-to-end learning, which means that raw CXR images were directly inputted into a deep learning model (EfficientNet v2-M) to extract their meaningful features in identifying disease categories;
- We experimented with our proposed method on the publicly available benchmark data set of the U.S. National Institutes of Health (NIH) [16] for three classes of pneumonia, pneumothorax, and normal (no finding); and
- We also experimented on our in-house data set of the Cheonan Soonchunhyang University Hospital (SCH) [17] for four classes of pneumonia, pneumothorax, tuberculosis, and normal (healthy).

The remainder of the paper is organized as follows. Section 2 describes previous and related methods using CNN models via transfer learning techniques. Section 3 then details our proposed method, while Section 4 analyzes our results. Section 5 compares our experimental results with other methods; finally, Section 6 concludes our study.

## 2. Related Works

Hong et al. [11] implemented transfer learning on -six models (VGG19, DenseNet201, Vanilla EfficientNetB7, EfficientNetB7 + Preprocessing, EfficientNetB7 + Multi GAP, and EfficientNetB7 + Preprocessing + Multi GAP) to classify lung diseases (pneumonia, pneumothorax, and normal) on CXR images of the NIH data set. EfficientNetB7 achieved the best performance (accuracy: 85.32%, sensitivity: 77.97%, and specificity: 88.98%). Hong et al. [11] also implemented transfer learning on six models (VGG19, DenseNet201, Vanilla EfficientNetB7, EfficientNetB7 + Preprocessing, EfficientNetB7 + Multi GAP, and EfficientNetB7 + Preprocessing + Multi GAP) to classify lung diseases (pneumonia, pneumothorax, tuberculosis, and normal) on CXR images of the in-house data set. EfficientNetB7 achieved the best performance (accuracy: 96.10%, sensitivity: 92.20%, and specificity: 97.40%). Apostolopoulos et al. [22] implemented transfer learning on five models (VGG19, MobileNetV2, Inception, Xception, and Inception ResNetV2) to classify lung diseases (pneumonia, COVID-19, and normal) on CXR images of the in-house data set. The VGG19 achieved the best performance (accuracy: 93.48%, sensitivity: 92.85%, and specificity: 98.75%). Yimer et al. [23] implemented transfer learning on Xception models to classify lung diseases (tuberculosis, pneumonia, COPD, pneumothorax, lung cancer, and normal) on CXR images of the NIH and in-house data sets. Xception achieved the best performance (accuracy: 97.30%, sensitivity: 97.20%, and specificity: 99.40%). Liu et al. [24] implemented transfer learning on six models (DenseNet121, InceptionV3, NASNet, ResNet50, VGG16, and Xception) to classify lung diseases (tuberculosis and normal) on CXR images of the in-house data set. DenseNet121 achieved the best performance (accuracy: 83.50%, sensitivity: 82.20%, and specificity: 84.90%).

Zak et al. [25] proposed a two-step DL method to classify lung diseases (pneumonia vs. tuberculosis) on CXR images of the in-house Shenzhen data set. The first step was to segment the lung region of interest (ROI) using U-Net and then apply transfer learning on three models (VGG16, ResNet-50, and InceptionV3), for which the pre-trained weights were from ImageNet. InceptionV3 achieved the best performance (accuracy: 82.00%, sensitivity: 82.33%, and specificity: 82.00%). Tian et al. [26] proposed a two-step DL method to classify lung diseases (pneumothorax vs. normal) on CXR images of the NIH and in-house SAHZUSM data sets. The first step was to segment the lung ROI using U-Net and then apply transfer learning on six models (Inception-ResNetV2, DenseNet, InceptionV3, VGG, NasNet, and ResNet), for which the pre-trained weights were from ImageNet. The Inception-ResNetV2 achieved the best performance (accuracy: 97.30% and specificity: 97.20%).

El Asnaoui et al. [27] implemented transfer learning on seven models (VGG16, VGG19, DenseNet201, Inception-ResNetV2, InceptionV3, ResNet50, and MobileNetV2) to classify lung diseases (pneumonia, corona virus, COVID-19, and normal) on CXR and CT images of the in-house data set. Inception-ResNet v2 achieved the best performance (accuracy: 92.18%, sensitivity: 92.11%, and specificity: 96.06%).

## 3. Materials and Methods

### 3.1. Data Set

We collected CXR images from the U.S. National Institutes of Health (NIH) [16] for three classes of pneumonia, pneumothorax, and normal (no finding) and split these images into training and validation sets, as shown in Table 1.

**Figure 1:**
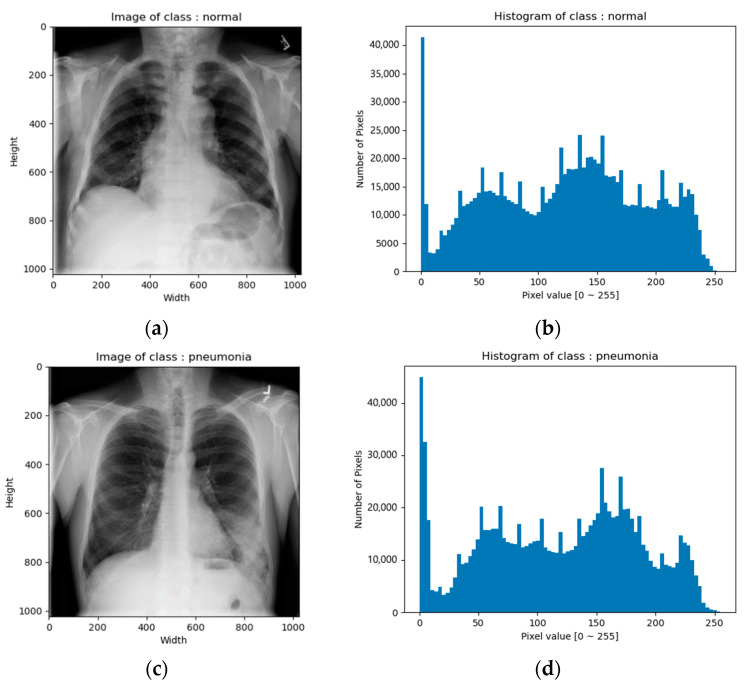

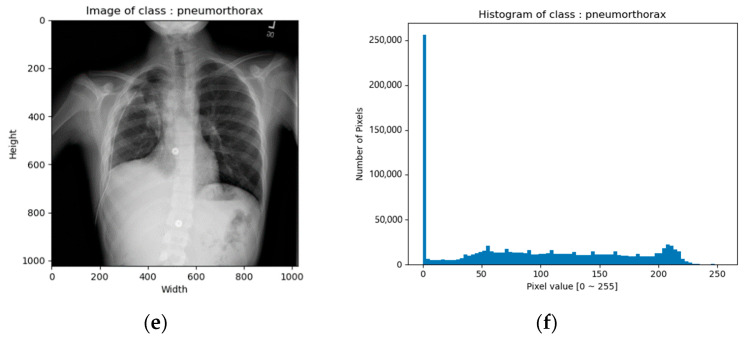
CXR images of NIH data set: (**a**) image of normal class; (**b**) histogram of normal class; (**c**) image of pneumonia class; (**d**) histogram of pneumonia class; (**e**) image of pneumothorax class; (**f**) histogram of pneumothorax class.

CXR images of the NIH data set were in png format. The image size was 1024 × 1024 and the bit depth was 8 bits [0–255]. Figure 1 shows example images and their corresponding histograms.

We also collected CXR images from the Cheonan Soonchunhyang University Hospital (SCH) [17] for four classes of pneumonia, pneumothorax, tuberculosis, and normal (healthy), and split these images into training, validation, and testing sets, as shown in Table 2.

**Figure 2:**
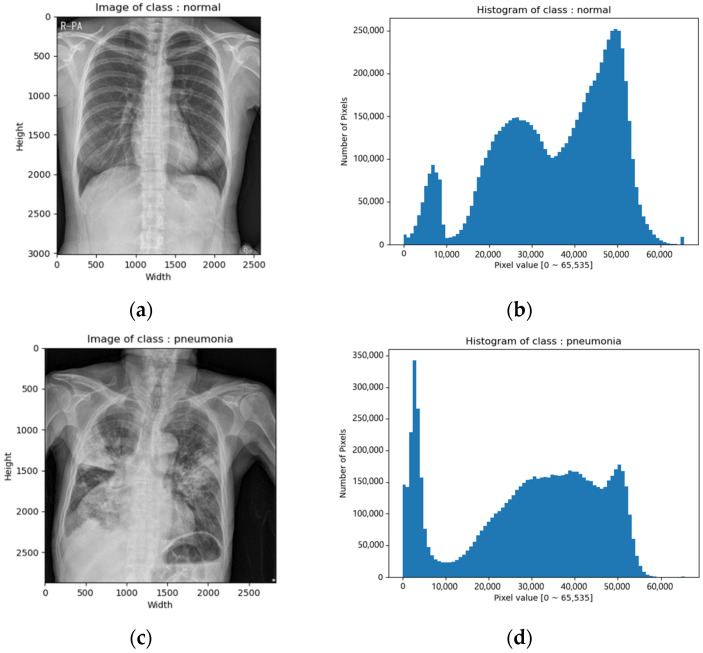

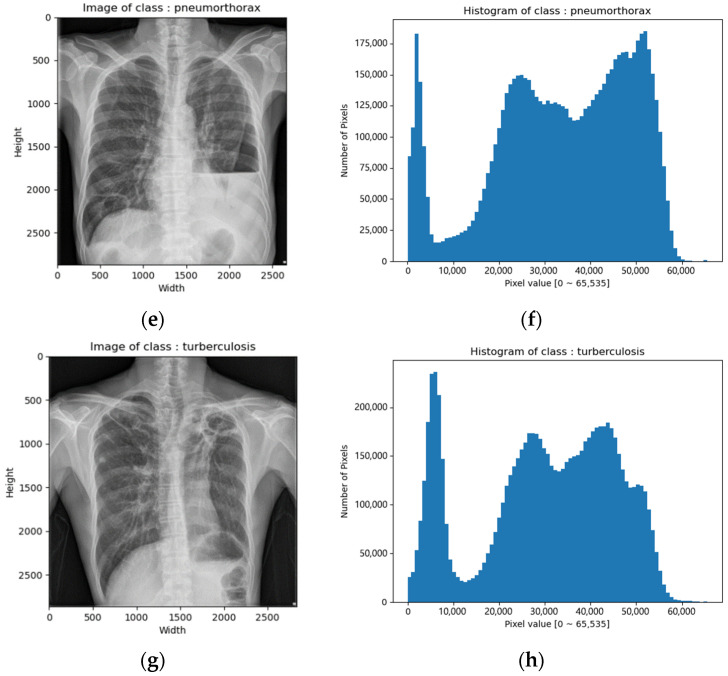
CXR images of SCH data set: (**a**) image of normal class; (**b**) histogram of normal class; (**c**) image of pneumonia class; (**d**) histogram of pneumonia class; (**e**) image of pneumothorax class; (**f**) histogram of pneumothorax class; (**g**) image of tuberculosis class; (**h**) histogram of tuberculosis class.

CXR images of the SCH data set were in tiff format. The image size varied from 2000 × 2000 to 3000 × 3000 and the bit depth was 16 bits [0–65,535]. Figure 2 depicts example images and their corresponding histograms.

### 3.2. Data Preprocessing

#### 3.2.1. Cropping and Resizing

**Figure 3:**
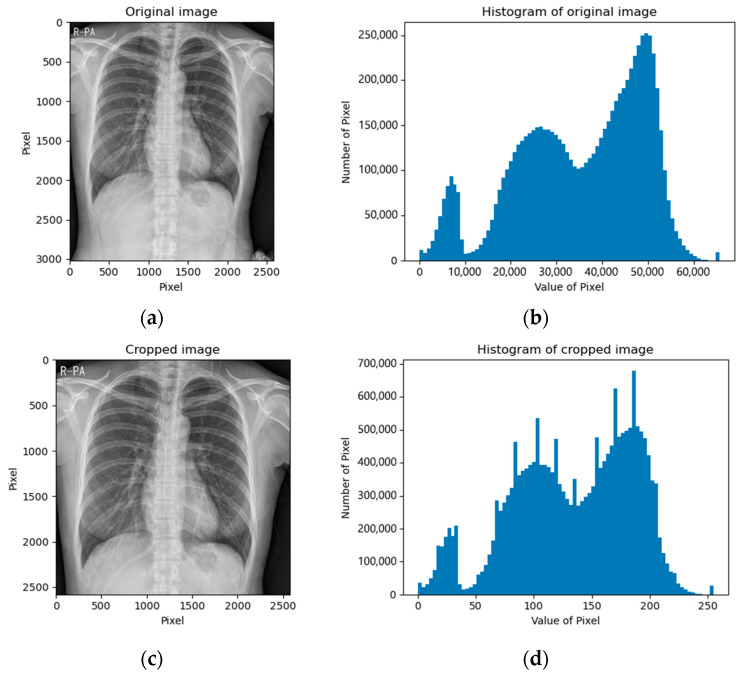
Cropped CXR images of the SCH data set: (**a**) original image; (**b**) histogram of original image; (**c**) bottom-cropped image; (**d**) histogram of bottom-cropped image.

The CXR images of the NIH data set were already in a square shape (1024 × 1024); thus, we kept the images as they were. The CXR images of the SCH data set varied in shape; thus, we cropped the images into square shapes by removing the bottom part because the bottom region mostly contained the belly part, which contained meaningless information. Figure 3 depicts examples of cropped images and their corresponding histograms.

Next, the CXR images from both the NIH and SCH were scaled to 688 × 688. Then, we performed a center cropping, making the images into a 600 × 600 size, which was an 87.5% cropping. Finally, they were converted into three-channel grayscale images by repeatedly tripling the information: 600 × 600 × 3.

#### 3.2.2. Data Augmentation

**Figure 4:**
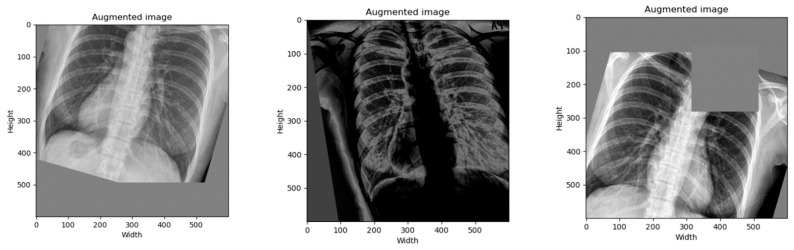
Augmented images were generated randomly using the RandAugment technique.

We performed a data augmentation to increase the number of samples and to diversify the image in position, orientation, brightness, and more. By the time of writing this paper, the best state-of-the-art data augmentation technique was RandAugment [28]. Therefore, we only applied it on the training data set by setting hyperparameters as follows: N = 5 and M = 20. Figure 4 depicts examples of augmented images.

Next, we concatenated the original images by the augmented images. Therefore, the number of images in the training set was increased twice, as shown in Table 3. Finally, we normalized the pixels of the images into [−1, +1].

### 3.3. Model

**Figure 5:**
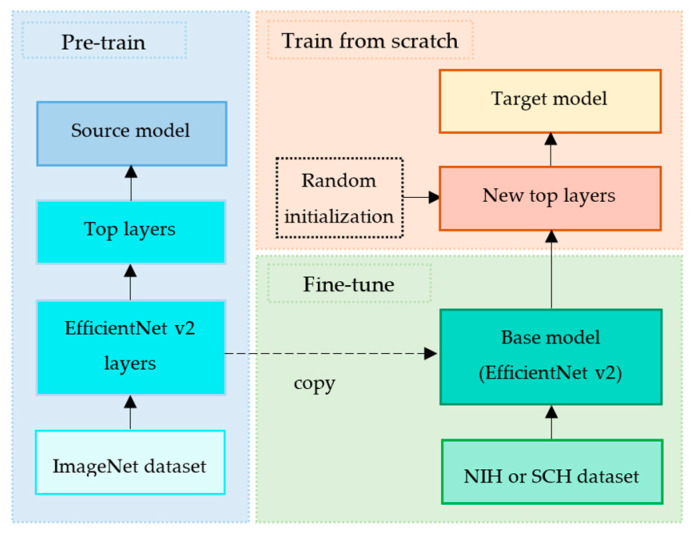
Our proposed transfer learning pipeline.

We implemented a transfer learning technique by exploiting EfficientNet v2 [29] as a base model and by defining the new top layers as a fine-tuning model. The common pipeline of transfer learning is firstly to extract features from the source data set and then fine-tune on the target data set [30]. Figure 5 depicts our proposed transfer learning pipeline.

#### 3.3.1. Pre-Train

EfficientNet v2 [29] is a deep convolutional neural network that was proven to outperform on the ImageNet data set [31], as shown in Table 4. EfficientNet v2 was trained on the ImageNet data set, and its learned weights were publicly shared. Inspired by its fast training speed and parameter efficiency, we exploited EfficientNet v2-M and its learned weights without its top layers as our base model. This process is called pre-train, as shown in Figure 5.

#### 3.3.2. Fine-Tune

Since the NIH and SCH data sets were much smaller than ImageNet data set, the fine-tuning process helped to improve our target models’ generalization ability, as shown in Figure 5. This fine-tuning copied all EfficientNet v2-M model designs and their parameters without the top layers. These parameters contained the knowledge learned from the ImageNet data set, which helped to identify shapes, patterns, edges, and object composition. We assumed that these general and similar features would also be effective for classifying lung diseases in CXR images.

#### 3.3.3. Train from Scratch

Since the ImageNet data set contained 1000 classes, the original EfficientNet v2 model was composed of the top layers to classify 1000 classes accordingly. However, our target classes were three classes (normal, pneumonia, and pneumothorax) of the NIH data set and four classes (normal, pneumonia, pneumothorax, and tuberculosis) of the SCH data set. Therefore, we defined the new top layers to fine-tune the extracted features into our target model.

Firstly, we trained our new top layers from scratch with a random initialize weight on the NIH or SCH data set, as shown in Table 5. The input layer had a shape of 600 × 600 × 3. Then, the input tensor passed to the base model (EfficientNet v2-M). The base model convoluted the input tensor into the last layer (before the top layer) with a shape of 19 × 19 × 1280, which produced 53,150,388 parameters. Next, we aggregated all the features using global average pooling 2D into 1280 features. Then, the dropout layer (rate was 0.4) was applied to avoid the overfitting issue. Finally, we aggregated the 1280 features into our target classes (three classes for NIH data set or four classes for SCH data set), which produced 3843 or 5124 parameters, respectively. We froze the base model (the base model was not trained). Thus, only 3843 or 5124 parameters were trained. This means that only 3843 or 5124 parameters were updated via gradient descent to minimize the loss during training. Meanwhile, 53,150,388 parameters were not trained and were updated by the entire model only during the forward pass.

Next, we trained a complete model (the base model and new top layers) on the NIH or SCH data set by enabling the base model to unfreeze, as shown in Table 6. This helped to achieve incremental improvements by re-training the pre-trained features with new top layers on our data sets (NIH or SCH data set). We set the training option of the batch normalization layers of the base model to False so that they were not trained. This helped to prevent damage to the features learned by the model so far. Thus, 52,862,199 or 52,863,480 parameters were re-trained while 292,032 were not.

### 3.4. Train

We conducted an experiment on a computer with an Intel CPU i9-9940X 3.30 GHz, 64-GB RAM, NVidia Geforce RTX 3090 (1755 MHz, 10,496 cores, 24 GB). The code was written in Python 3.9 and Tensorflow 2.8 with CUDA 11.2 and cuDNN 8.

Since the data of each class had a different number of samples, we defined a class weight on the training set, as shown in Table 7. The class weights helped to balance the data samples by assigning a low weight to large samples and a high weight to small samples [32]. The assigned class weights, *C*, were calculated as follows:

(1) $$C_{i}=\frac{\sum_{i}^{N}S_{i}}{N\times S_{i}},$$

where N is the number of classes (N=3 for the NIH data set or N=4 for the SCH data set) and $S_{i}$ is the number of samples of class i.

We applied the Lookahead optimizer [33] to wrap the Rectified Adam optimizer [34]. This wrapping improved learning stability by helping the Rectified Adam optimizer escape from local minima and lowering the loss variance. Additionally, we applied a warm-up by setting the warm-up proportion to 0.1. Since we had two trainings, the learning rates were set as follows.

(1) Train on the new top layers: We set an initial learning rate of 1e-3 and a minimum learning rate of 1e-4 because the new top layers were small. We trained these layers for 50 epochs.
(2) Train on the complete model: We set the initial learning rate of 1e-4 and minimum learning rate of 1e-6 because the base model was very large. Thus, the very low learning rate helped to prevent overfitting when re-training on our small data set. We trained it for 25 epochs.

In both trainings, the clip norm of the Rectified Adam optimizer was set to 1.0. This gradient clipping helped to avoid exploding gradients [35]. Additionally, we applied the categorical cross-entropy loss, for which its label smoothing was set to 0.1 [36]. Table 8 summarizes our empirical hyperparameters.

## 4. Results

### 4.1. Evaluation Metrics

We followed the evaluation metrics of a classification study. We used accuracy (Acc) to evaluate the overall quality of our classification. Since our number of samples was imbalanced, we additionally used sensitivity (Sen) to evaluate the quality of positive prediction and specificity (Spe) to evaluate the quality of negative prediction. They were calculated as follows.

(2) $$Acc=\frac{TP+TN}{TP+TN+FP+FN}\;,$$

(3) $$Sen=\frac{TP}{TP+FN},$$

(4) $$Spe=\frac{TN}{TN+FP},$$

where TP, FP, TN, and FN represent true positive, false positive, true negative, and false negative, respectively.

### 4.2. Three-Class Classification

**Figure 6:**
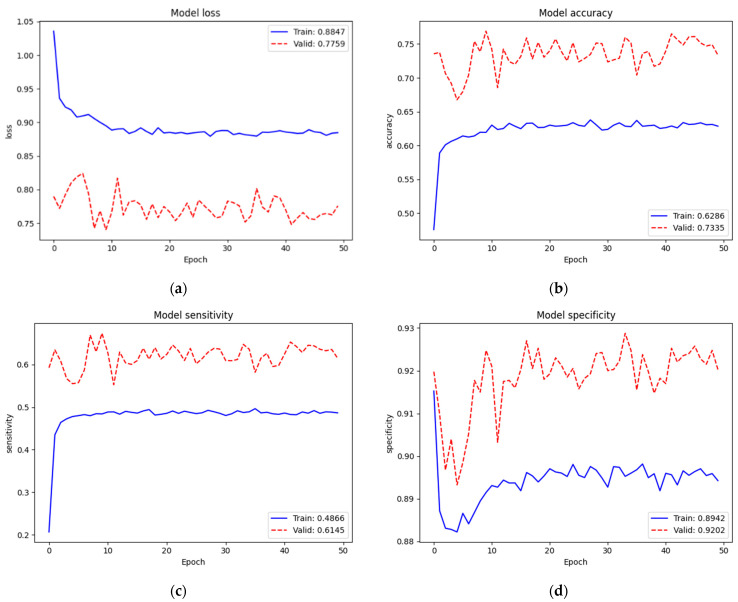
Train and validation performances of the NIH data set on new top layers: (**a**) loss; (**b**) accuracy; (**c**) sensitivity; (**d**) specificity.

Figure 6 depicts the training and validation performance of the NIH data set on the new top layers. Loss, accuracy, sensitivity, and specificity of training were 0.8847, 62.86%, 48.66%, and 89.42%, respectively. Since we trained on very small parameters (3843 parameters) from scratch, the training performance was low. Additionally, we noticed that the validation performance (Loss = 0.7759, Acc = 73.35%, Sen = 61.45%, and Spe = 92.02%) was better than the training performance. We assumed that this case occurred because of regularization mechanisms. When training, the features were set to zero by 40% since we used dropout (rate = 0.4); however, all features were fully used when validating. Additionally, even after concatenating the original and augmented images, our training set (16,000 images) was still small compared to the model network (EfficientNet v2-M). However, we found that this was not an issue due to this training not being our final target. Conversely, it helped the new top layers to tune in to our target model well at the next training.

**Figure 7:**
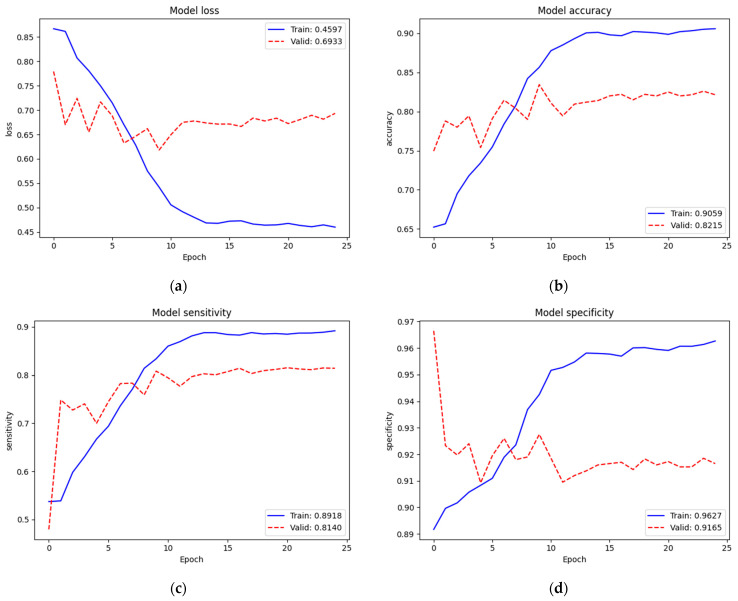
Train and validation performances of the NIH data set on the complete model: (**a**) loss; (**b**) accuracy; (**c**) sensitivity; (**d**) specificity.

To identify the three classes of lung diseases, the validation performance (Loss = 0.7759, Acc = 73.35%, Sen = 61.45%, and Spe = 92.02%) of only the new top layers was not so bad but also not yet acceptable. Therefore, we re-trained (second train) on the complete model. Figure 7 depicts the training and validation performances of the NIH data set on the complete model. The loss, accuracy, sensitivity, and specificity of training were 0.4597, 90.59%, 89.18%, and 96.27%, respectively. Since we trained on the complete model excluding batch normalization layers (52,862,199 parameters) from the pre-trained weights of ImageNet and our recently trained new top layers, the train performance was improved. The validation performance (Loss = 0.6933, Acc = 82.15%, Sen = 81.40%, and Spe = 91.65%) was also improved. In this training, we noticed that the dropout mechanism did not cause a validation performance higher than the training one, which expressed that it was trained and fit the model well. Additionally, we noticed that the loss started to converge after epoch 15. Thus, we assumed that 25 epochs were enough for this training.

Table 9 shows a summary of the validation performance of the NIH data set on the first train (train from scratch on our new top layers) and the whole train (second train) on the complete model. The performance of the second train was improved acceptably.

**Figure 8:**
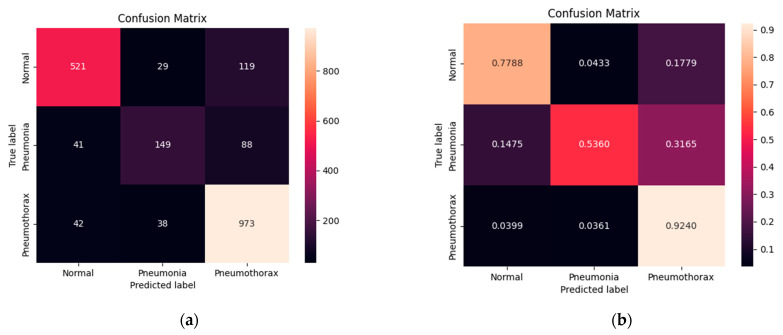
Confusion matrix of the pneumonia, pneumothorax, and normal classes of the validation set of the NIH data set: (**a**) numerical; (**b**) percentage.

Figure 8 shows the confusion matrix of normal, pneumonia, and pneumothorax classes of the validation set of the NIH data set. Our model correctly predicted for the normal class 521 images (77.88%) out of 669 images while it confused as pneumonia 29 images (4.33%) and pneumothorax 119 images (17.79%). It correctly predicted for the pneumonia class 149 images (53.60%) out of 278 images while it confused as normal 41 images (14.75%) and pneumothorax 88 images (31.65%). Finally, it correctly predicted for the pneumothorax class 973 images (92.40%) out of 1053 images while it confused as normal 42 images (3.99%) and pneumonia 38 images (3.61%). We found that our model predicted for pneumothorax better than the others.

**Figure 9:**
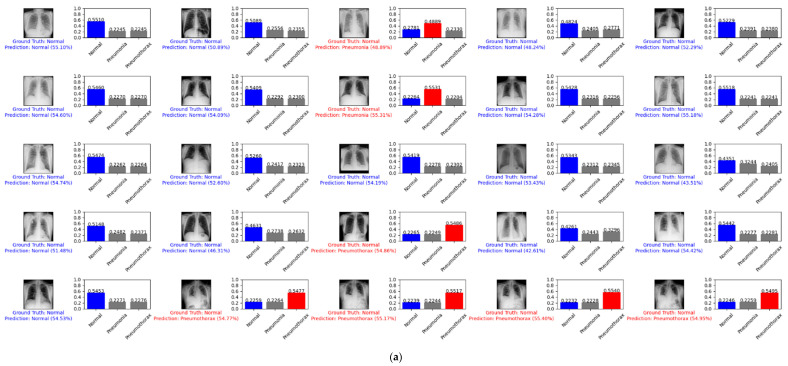

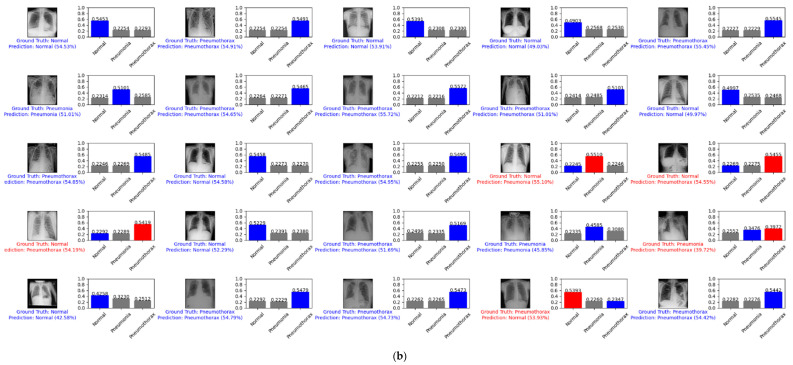
Example predictions on the normal, pneumonia, and pneumothorax classes of the validation set of the NIH data set: (**a**) the first 25 images of the normal class; (**b**) 25 random images.

Figure 9 depicts example predictions for the normal, pneumonia, and pneumothorax classes of the validation set of the NIH data set. The true label is shown in blue. If our prediction was correct, it will still show in blue with its confidence score. Otherwise, if our prediction was wrong, it will show in red. We predicted the first 25 images of the normal class and demonstrated their prediction scores, as shown in Figure 9a. There were 18 images that were correctly predicted (blue color) and seven images that were wrong (red color). We also predicted another 25 random images of the validation set, as shown in Figure 9b. There were 20 images that were correctly predicted and five images that were wrong.

### 4.3. Four-Class Classification

**Figure 10:**
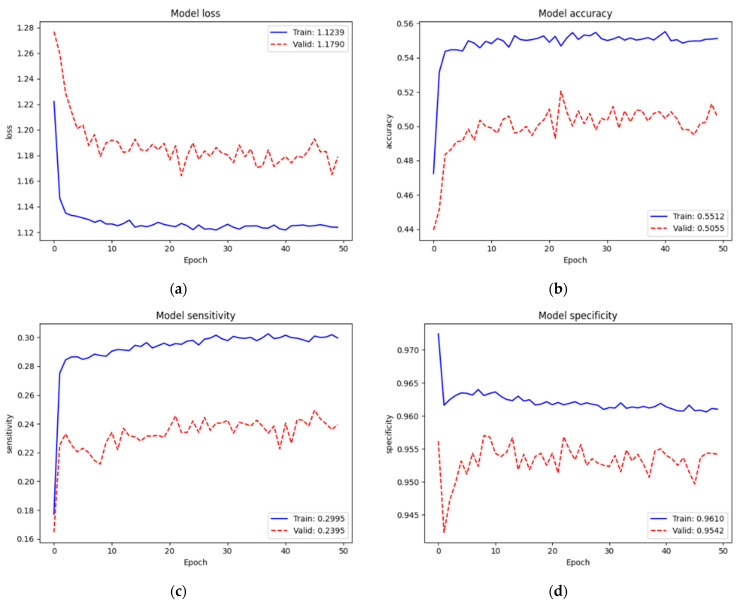
Training and validation performance of the SCH data set on new top layers: (**a**) loss; (**b**) accuracy; (**c**) sensitivity; (**d**) specificity.

Figure 10 depicts the training and validation performance of the SCH data set on new top layers. Loss, accuracy, sensitivity, and specificity of training were 1.1239, 55.12%, 29.95%, and 96.10%, respectively. We still faced the same problem of low performance since we trained on very small parameters (5124) from scratch. However, we did not have the issue of applying dropout regularization since the validation performance (Loss = 1.1790, Acc = 50.55%, Sen = 29.95%, and Spe = 95.42%) was lower than the training performance. We assumed that this was because our training set (58,564 images) was enough for a model network (EfficientNet v2-M). Even though the validation performance was low, we found that it was not an issue; also, this training was not our final target. Conversely, it helped the new top layers to tune in to our target model well at the next training.

**Figure 11:**
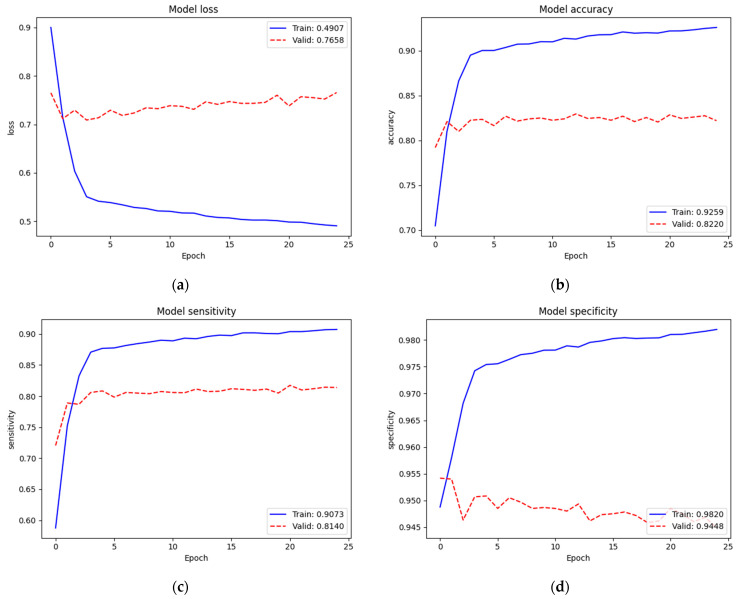
Training and validating performance of the SCH data set on the complete model: (**a**) loss; (**b**) accuracy; (**c**) sensitivity; (**d**) specificity.

We re-trained (second train) on the complete model. Figure 11 depicts the training and validation performances of the SCH data set on the complete model. Loss, accuracy, sensitivity, and specificity of training were 0.4907, 92.59%, 90.73%, and 98.20%, respectively. Since we trained on the complete model excluding the batch normalization layers (52,863,480 parameters) from the pre-trained weights of ImageNet and our recently trained new top layers, the train performance was improved. Validation performance (Loss = 0.7658, Acc = 82.20%, Sen = 81.40%, and Spe = 94.48%) was also improved. In this training, the number of classes increased to four. However, since we also increased the sample numbers of the training set, we could maintain the performance.

Table 10 summarizes the validation performance of the SCH data set on the first training (from scratch on our new top layers) and the whole training (second training) on the complete model. The performance of the second training was acceptably improved.

**Figure 12:**
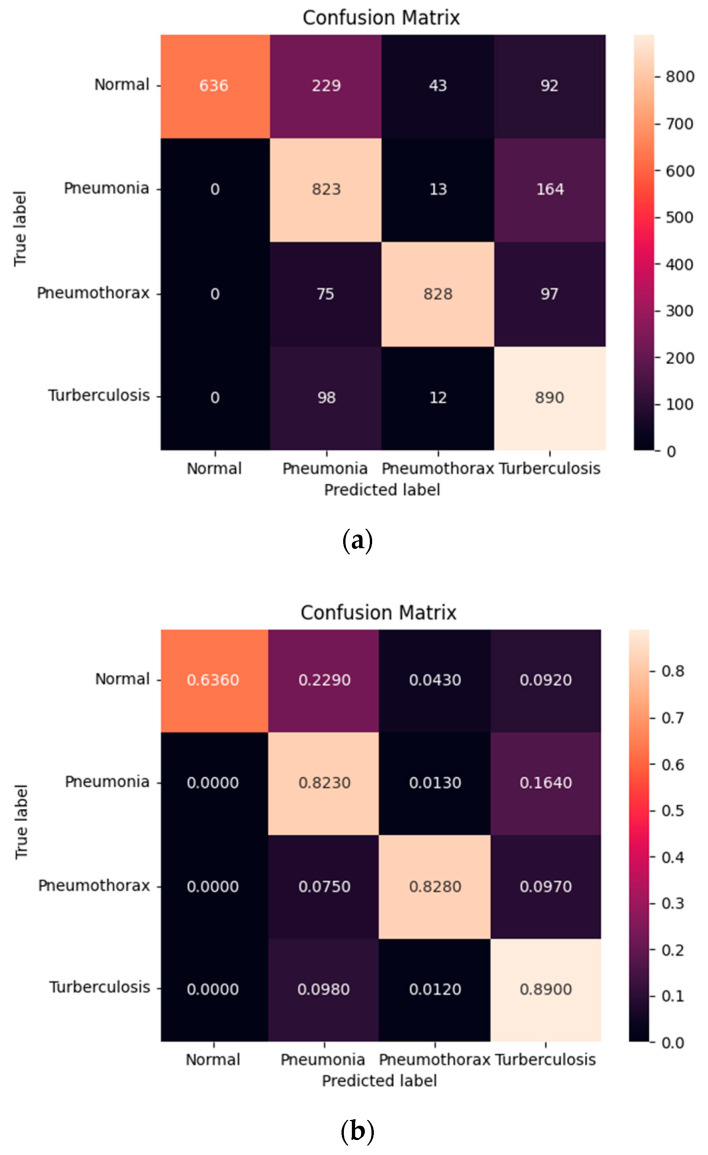
Confusion matrix of the Pneumonia, Pneumothorax, Tuberculosis, and Normal class of testing set of the SCH data set: (**a**) numerical; (**b**) percentage.

Figure 12 shows the confusion matrix of the normal, pneumonia, pneumothorax, and tuberculosis classes of the testing set of the SCH data set. Our model correctly predicted for the Normal class 636 images (63.60%) out of 1000 images while it confused as pneumonia 229 images (22.90%), pneumothorax 43 images (4.30%), and tuberculosis 92 images (9.20%). It correctly predicted for the pneumonia class 823 images (82.30%) out of 1000 images while it confused as normal 0 image (0%), pneumothorax 13 images (1.30 %), and tuberculosis 164 images (16.40%). It correctly predicted for the pneumothorax class 828 images (82.80%) out of 1000 images while it confused as normal 0 image (0%), pneumonia 75 images (7.50%), and tuberculosis 97 images (9.70%). Finally, it correctly predicted for the tuberculosis class 890 images (89.90%) out of 1000 images while it confused as normal 0 image (0%), pneumonia 98 images (9.80%), and pneumothorax 12 images (1.20%). We found that our model predicted for tuberculosis better than the others and expressed that those three classes of diseases were not confused with the normal class.

**Figure 13:**
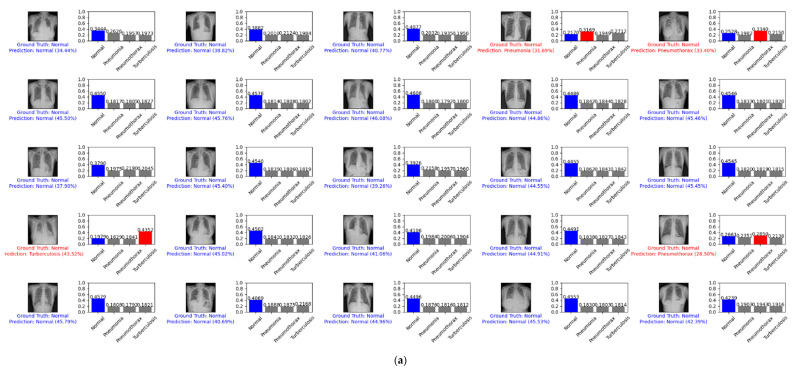

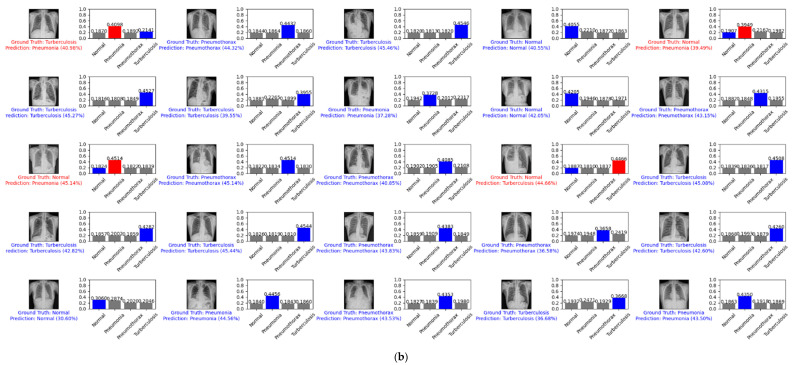
Example predictions for the normal, pneumonia, pneumothorax, and tuberculosis classes of the testing set of the SCH data set: (**a**) the first 25 images of the normal class; (**b**) 25 random images.

Figure 13 depicts an example prediction for the normal, pneumonia, pneumothorax, and tuberculosis classes of the testing set of the SCH data set. The true label is shown in blue. If our prediction was correct, it will still show in blue with its confidence score. Otherwise, if our prediction was wrong, it will show in red. We predicted the first 25 images of the normal class and demonstrated their prediction scores, as shown in Figure 13a. There were 21 images that were correctly predicted (blue color) and four images that were wrongly predicted (red color). We also predicted another 25 random images of the testing set, as shown in Figure 13b. There were 21 images that were correctly predicted and four images that were wrongly predicted.

## 5. Discussion

### 5.1. Experimental Analysis

We followed an experimental analysis of a DL design and algorithm study [37,38]. We conducted an experiment with a vanilla method of three CNN models such as VGG19, DenseNet201, and EfficientNet B7 on the same data of NIH data set. This experiment was to compare our proposed method and a vanilla method of three CNN models. Table 11 shows the performance comparison of a three-class classification (normal, pneumonia, and pneumothorax). The VGG19 model achieved validation performance as Acc = 62.40%, Sen = 62.58%, and Spe = 62.03%. The DenseNet201 model achieved validation performance as Acc = 30.45%, Sen = 15.92%, and Spe = 59.34%. The EfficientNet B7 model achieved validation performance as Acc = 60.45%, Sen = 63.86%, and Spe = 53.66%. The EfficientNet v2-M model achieved validation performance as Acc = 82.15%, Sen = 81.40%, and Spe = 91.65%. We can see that the DenseNet201 model achieved the lowest performance while EfficientNet v2-M achieved the highest performance. Therefore, we can assume that our proposed method with EfficientNet v2-M model is the best choice among those vanilla models in lung diseases’ classification.

We conducted the experimental analysis only on the NIH data set. After we figured out that our proposed method with EfficientNet v2-M model outperformed other CNN models, we did not conduct the experimental analysis on the SCH data set because we assumed that our proposed method would still outperform the others on the SCH data set. Therefore, we experimented with our proposed method with the EfficientNet v2-M model on the SCH data set only.

### 5.2. Method Comparison

Table 12 shows the performance comparison on the multi-class classification. We can see that some papers [25,26] used the two-step method. First, they segmented the ROI of the lung region using U-Net and then classified them using InceptionV3 and Inception-ResNetV2, respectively. This two-step method is costly in both time and effort. It requires a data set for segmentation and classification. Additionally, it strongly relies on the segmentation model. If the performance of segmentation is low, it will yield low performance on classification, too. Another paper [27] used two types of data (CXR and CT). This method requires a lot of data set collection. Other papers [11,22,23,24] used one type of data (CXR) and a one-step method that was similar to our proposed method. However, it is hard to compare the performance since they used a different number of classes and data sets. Only one paper [11] conducted the same configuration as ours on the NIH data set.

Table 13 shows the performance comparison on multi-class classification on the NIH data set between Hong et al. [11] and ours. We can see that Hong et al.’s [11] outperformed ours on accuracy. However, since the number of samples was imbalanced, only accuracy did not express the performance precisely. Therefore, we additionally used sensitivity (Sen) to evaluate the quality of positive prediction, and specificity (Spe) to evaluate the quality of negative prediction. We can see that our method outperformed that of Hong et al. [11] on sensitivity and specificity, respectively. This means that, among positive images, our model correctly predicted 81.40%. Additionally, among negative images, our model correctly predicted 91.65%.

## 6. Conclusions

In this paper, we proposed the EfficientNet v2-M model using the pre-trained weights of ImageNet to classify lung diseases on CXR images to improve the efficiency and accuracy of CADs’ diagnostic performance. Our proposed method was a one-step, end-to-end learning. Firstly, the data were augmented to increase the number of samples and variance diversities. Then, they were directly inputted into a deep learning model to extract their meaningful features in identifying disease categories. In the transfer learning process, the first training was conducted on our new, defined top layers and the second training was conducted on the entire model.

Our method predicted for the three classes of normal, pneumonia, and pneumothorax of the NIH data set and achieved validation performances of Loss = 0.6933, Acc = 82.15%, Sen = 81.40%, and Spe = 91.65%. Our method predicted for the four classes of normal, pneumonia, pneumothorax, and tuberculosis of the SCH data set and achieved validation performances of Loss = 0.7658, Acc = 82.20%, Sen = 81.40%, and Spe = 94.48%. The testing accuracies for the normal, pneumonia, pneumothorax, and tuberculosis classes were 63.60%, 82.30%, 82.80%, and 89.90%, respectively.

We learned that classifying multi-class lung diseases on CXR images (grayscale image) is very challenging without data augmentation and transfer learning. Additionally, we also figured out that the regularization mechanism (dropout) posed a trade-off between losing a great deal of meaningful information and overfitting. If we give a high dropout rate, we will face losing a lot of important features. On the other hand, if we give a low rate, the model will fit well on the training set but not the validation or testing set. Finally, we empirically and optimally tuned our hyperparameters to achieve a significant performance on multi-class lung diseases’ classification.

## Figures and Tables

**Table 1:** NIH data set.

Class	Training Set	Validation Set	Total
Normal	2676	669	3345
Pneumonia	1114	278	1392
Pneumothorax	4210	1053	5263
Total	8000	2000	10,000

**Table 2:** SCH data set.

Class	Training Set	Validation Set	Testing Set	Total
Normal	10,203	500	1000	11,703
Pneumonia	7080	500	1000	8580
Pneumothorax	4631	500	1000	6131
Tuberculosis	7368	500	1000	8868
Total	29,282	2000	4000	35,282

**Table 3:** Combination samples of the original and augmented data of the training set.

Data Set	Original Set	Augmented Set	Total
NIH	8000	8000	16,000
SCH	29,282	29,282	58,564

**Table 4:** Family models of EfficientNet v2 training on ImageNet.

Model	Accuracy	Param #	FLOPs	Inference Time	Train Time
EfficientNet v2-S	84.90%	22 M	8.8 B	24 ms	9 ms
EfficientNet v2-M	86.20%	54 M	24 B	57 ms	15 ms
EfficientNet v2-L	86.80%	120 M	53 B	98 ms	26 ms
EfficientNet v2-XL	87.30%	208 M	94 B	-	45 ms

Param # refers to number of parameters.

**Table 5:** Our target model trained on the new top layers of the NIH or SCH data set.

Layer	Output Shape	Param #
Input	(None, 600, 600, 3)	0
Efficientnetv2-m	(None, 19, 19, 1280)	53,150,388
Global_average_pooling2d	(None, 1280)	0
Dropout	(None, 1280)	0
Dense	(None, 3) or (None, 4)	3843 or 5124
Total params: 53,154,231 or 53,155,512		
Trainable params: 3843 or 5124		
Non-trainable params: 53,150,388		

Param # refers to number of parameters.

**Table 6:** Our target model trained on a complete model (base model and new top layers) of the NIH or SCH data set.

Layer	Output Shape	Param #
Input	(None, 600, 600, 3)	0
Efficientnetv2-m	(None, 19, 19, 1280)	53,150,388
Global_average_pooling2d	(None, 1280)	0
Dropout	(None, 1280)	0
Dense	(None, 3) or (None, 4)	3843 or 5124
Total params: 53,154,231 or 53,155,512		
Trainable params: 52,862,199 or 52,863,480		
Non-trainable params: 292,032		

Param # refers to number of parameters.

**Table 7:** Class weights of the training set of the NIH and SCH data sets.

Data Set	Class	Training Set	Class Weight
NIH	Normal	5352	0.9965
	Pneumonia	2228	2.3937
	Pneumothorax	8420	0.6334
SCH	Normal	20,406	0.7537
	Pneumonia	14,160	1.0280
	Pneumothorax	9262	1.4387
	Tuberculosis	14,736	0.9946

**Table 8:** Summary of our empirical hyperparameters.

Parameters	Value
Image shape	600 × 600 × 3
Pixel normalization	[−1, +1]
Data augmentation	RandAugment (N = 5, M = 20)
Base model	EfficientNet v2-M (pre-trained weights = ImageNet)
Model regular	Dropout (rate = 0.4)
Model optimizer	Lookahead, Rectified Adam (clip norm = 1)
Warm-up proportion	0.1
Learning rate	1st train = [1e-3, 1e-4], 2nd train = [1e-4, 1e-6]
Loss	Categorical cross entropy (label smoothing = 0.1)
Classifier	Softmax
Class	NIH data set = 3, SCH data set = 4
Epoch	1st train = 50, 2nd train = 25
Batch size	8

**Table 9:** Summary of the validation performance of the NIH data set.

Train	Loss	Accuracy	Sensitivity	Specificity
Train from scratch (1st train)	0.7759	73.35%	61.45%	92.02%
Whole train (2nd train)	0.6933	82.15%	81.40%	91.65%

**Table 10:** Summary of the validation performance of the SCH data set.

Train	Loss	Accuracy	Sensitivity	Specificity
Train from scratch (1st train)	1.1790	50.55%	29.95%	95.42%
Whole train (2nd train)	0.7658	82.20%	81.40%	94.48%

**Table 11:** Performance comparison of three-class classification on validation set of NIH data set (%).

Method	Model	Accuracy	Sensitivity	Specificity
Vanilla	VGG19	62.40	62.58	62.03
Vanilla	DenseNet201	30.45	15.92	59.34
Vanilla	EfficientNet B7	60.45	63.86	53.66
Ours	EfficientNet v2-M	**82.15**	**81.40**	**91.65**

Bold face represents higher score in column.

**Table 12:** Performance comparison on multi-class classification.

Paper	Data Set	Image	Class	Step #	Model	Performance
[25]	In-house	CXR	Pneumonia, Tuberculosis	2	U-Net, InceptionV3	Acc = 82.00%, Sen = 82.33%, Spe = 82.00%
[26]	NIH, In-house	CXR	Pneumothorax, Normal	2	U-Net, Inception-ResNetV2	Acc = 97.30%, Spe = 97.20%
[27]	In-house	CXR, CT	Pneumonia, Corona virus, COVID-19, Normal	1	Inception-ResNetV2	Acc = 92.18%, Sen = 92.11%, Spe = 96.06%
[22]	In-house	CXR	Pneumonia, COVID-19, Normal	1	VGG19	Acc = 93.48%, Sen = 92.85%, Spe = 98.75%
[23]	NIH, In-house	CXR	Tuberculosis, Pneumonia, COPD, Pneumothorax, Lung cancer, Normal	1	Xception	Acc = 97.30%, Sen = 97.20%, Spe = 99.40%
[24]	In-house	CXR	Tuberculosis, Normal	1	DenseNet121	Acc = 83.50%, Sen = 82.20%, Spe = 84.90%
[11]	NIH	CXR	Pneumonia, Pneumothorax, Normal	1	EfficientNet B7	Acc = 85.32%, Sen = 77.97%, Spe = 88.98%
[11]	In-house	CXR	Pneumonia, Pneumothorax, Tuberculosis, Normal	1	EfficientNet B7	Acc = 96.10%, Sen = 92.20%, Spe = 97.40%
Ours	NIH	CXR	Pneumonia, Pneumothorax, Normal	1	EfficientNet v2-M	Acc = 82.15%, Sen = 81.40%, Spe = 91.65%
Ours	In-house	CXR	Pneumonia, Pneumothorax, Tuberculosis, Normal	1	EfficientNet v2-M	Acc = 82.20%, Sen = 81.40%, Spe = 94.48%

Step # refers to number of steps.

**Table 13:** Performance comparison on multi-class classification on the NIH data set (%).

Paper	Model	Accuracy	Sensitivity	Specificity
[11]	EfficientNet B7	**85.32**	77.97	88.98
Ours	EfficientNet v2–M	82.15	**81.40**	**91.65**

Bold face represents higher score in column.

## Data Availability

No new data were created in this study. Data sharing is not applicable.
